# Genotype × Environment Interaction for Wheat Yield Traits Suitable for Selection in Different Seed Priming Conditions

**DOI:** 10.3390/plants9121804

**Published:** 2020-12-19

**Authors:** Vera Popović, Nataša Ljubičić, Marko Kostić, Mirjana Radulović, Dragana Blagojević, Vladan Ugrenović, Dragana Popović, Bojana Ivošević

**Affiliations:** 1 Institute of Field and Vegetable Crops, 21000 Novi Sad, Serbia; 2 Biosense Institute, University of Novi Sad, 21000 Novi Sad, Serbia; mirjana.radulovic@biosense.rs (M.R.); dragana.blagojevic@biosense.rs (D.B.); bojana.ivosevic@biosense.rs (B.I.); 3 Faculty of Agriculture, University of Novi Sad, 21000 Novi Sad, Serbia; marko.kostic@polj.uns.ac.rs; 4 Institute for Soil Science, 11000 Belgrade, Serbia; vladan.ugrenovic@gmail.com; 5 Faculty of Economics in Subotica, University of Novi Sad, 21000 Novi Sad, Serbia; draaaganap@gmail.com

**Keywords:** wheat, zinc oxide nanoparticles, GEI, AMMI

## Abstract

Different seed priming treatments are widely used in order to improve the nutritional status of wheat, as well as to improve its grain yield and yield- related traits. The present study aimed to evaluate the impact of seed priming with zinc oxide nanoparticles (ZnO NPs) on the yield related traits, such as, field emergence, plant height, spike length and grain yield per plant of four winter wheat genotypes (*Triticum aestivum* L.) during two vegetation seasons of 2018/2019 and 2019/2020. The seeds of each wheat genotypes were primed with different concentrations of ZnO NPs (0 mg L^−1^, 10 mg L^−1^, 100 mg L^−1^ and 1000 mg L^−1^) for 48 h in a dark box by continuous aeration and were sown in soil pots with 60–70% moisture content until full maturity. The additive main effects and multiplicative interaction (AMMI) models were used to study the genotype environment effects. The results indicated that the plants response to ZnO nanoparticles significantly increased all of the observed traits of the wheat, while its maximum rates reduced the traits of the wheat. The AMMI analysis revealed the very complex nature of the variation observed in the trial and showed the significant effect of the G×E interaction, in which the first main component was significant for all components.

## 1. Introduction

Grown on more than 200 million hectares of land worldwide, wheat is now the most widely cultivated cereal in the world, as well as one of the most important crops for global food security [1]. In oreder to meet the growing demand from an increasing world’s population, there is a need to increase wheat productivity worldwide. For that purpose, the wheat yields have to go up by 15%, despite different climates and precipitation [2]. Wheat yield is a complex, polygenic trait and the result of the value of the yield components, such as plant height, the number of productive tillers, the number of grain spike per spike, the grain weight per spike, the thousand grain mass and other traits [3]. Since the increment in one yield component might have a positive or negative effect on the other components, a large number of studies have been conducted to investigate the genetic basis of these traits of wheat. Breeders frequently use yield components to improve the grain yield, despite the fact that these components compensate each other in practice and an increase in one causes a decrease in the other [4,5]. Recent studies suggest that seed priming methods possess a great potential the enhance growth quality, grain yield and yield related traits of wheat. Seed priming method has been shown to be prominent, simple, cost effective and beneficial especially under adverse environmental conditions [6]. Among the different priming solutions in use, zinc oxide nanoparticles (ZnO NPs) are the most widely used. It has been reported by several authors that seed priming with ZnO NPs could promote seed germination, improve zinc eficiencies, root volume, increase plant growth, yield traits, as a biomass, stem height and spike length in wheat [7,8]. Aside from its influence on crop growth, development and productivity, plant nutrition with Zn plays a key role in the germination, emergence of seedlings and establishment of population. These effects promoted by Zn are directly linked to the functions of this element in the plants protein synthesis, cell elongation, auxin biosynthesis, used in the gene expression, resistance to stresses and pollen formation [9,10,11]. Hence, the assessment of the impacts of ZnO NPs on the plant traits of wheat could provide new insights into the application of nanotechnology in improving the yield traits of wheat. However, although wheat is widely cultivated under wide ranges of climatic conditions, the stability performance of wheat genotypes in contrast environments is of great importance as this ensures reliable selections of genotypes with high yield and consistent performance for wide and specific environments [12,13]. Wheat yield depends on genetic and environmental factors and their interaction [3]. Differential genotypic responses to different environments are collectively called genotype-by-environment interaction (GEI) [14]. To study this effect, several techniques have been employed to estimate the effect of yield and yield related traits across different environments. The use of stability parameters was confirmed to exploit interaction effect of genotypes grown in diverse environment. The Additive Main Effect and Multiplicative Interaction model (AMMI) is one of the most widely used and powerful approaches to the analysis of genotype-by-environment interaction. It can be used to understand and structure interactions between genotypes and environments. The AMMI model combines analysis of variance (ANOVA) to test the main effects of both genotypes (G) and environments (E) and principal components analysis (PCA) to analyse the residual G × E interaction (GEI) component. It separates G, E and GEI as is required for most agricultural research purposes. AMMI is ordinarily the model of choice when the main effects and interactions are both important, which is the most common case with yield trials [15,16]. Applications of the AMMI model to yield trials have been used during the last two decades and there have been several recent review articles [16,17,18,19]. Although the main aim of this investigation was to follow the stability of genotypes, from the wheat breeding view it was very important to consider which wheat genotypes reacted favorably to seed priming treatments. Since the performance of wheat under different ZnO NPs seed priming treatments could be of great importance in assisting breeders and producers in increasing wheat productivity, the objectives of this study were to: (1) determine the relative contributions of the genotype, environment and their interaction in four important yield related traits of four wheat genotypes using AMMI models; (2) identify a high yielding and stable wheat genotype across different treatments and to; (3) identify a suitable genotypes for each treatment; 4) taking into consideration which varieties reacted favorably to seed priming.

## 2. Results and Discussion

Yield is a complex characteristic which is largely dependent on genetic potential and varies considerably primarily as a result of the environmental conditions during the growing season [20,21,22,23]. Grain yield can be improved since it is the result of many quantitative traits controlled by numerous genes, each having small effects, improving direct and some indirect components [22]. The assessment of the impacts of ZnO NPs on plant traits of wheat could provide new insights into the application of nanotechnology in improving the yield and yield-related traits of wheat. 

### 2.1. Field Emergence

The results of this investigation have indicated that the field emergence of wheat genotypes increased with the increasing ZnO NPs concentration applied.

The greatest overall mean value (99.11%) was denoted for wheat variety Futura (G4) in both season at 10 mg L^−1^ ZnO NPs was applied. At the same level of ZnO NPs, the maximum was established by genotype Pobeda (G1), while genotypes NS40S (G2) and NK Ingenio (G3) achieved their maximum the emergence at level of 100 mg L^−1^ ZnO NPs was applied. Low values of field emergence were observed in the control plants, whereas the lowest values were found at the maximum concentration of ZnO NPs (Table 1).

The presented results revealed that the different treatments influenced the differences in field emergence. The variability was caused by the variability of the genetic materials and mostly by the environmental conditions in which the wheat experiment was performed.

The seed priming technique is used to increase the viability of seed to increase its ability to grow under a wide range of environmental conditions or to achieve high, fast and homogeneous percentages of germination and field emergence and good field establishment [24]. The timing of the seedling emergence is an important factor in determining phenological development, growth and grain yield of wheat [25,26]. Seed emergence is influenced by a large number of processes, including agronomic factors, genetic factors and interactions between seeds and environmental conditions [26,27]. A higher percentage of germination and seedling growth of wheat with the application of ZnO NPs has been reported by Solanki and Laura [11]. Neto et al. [10] reported similar findings where ZnO NP application promotes an increase in maize germination and vigor up to a threshold concentration, after which, increasing concentrations provide diminishing benefit.

The combined ANOVA showed that field emergence of wheat was significantly affected by the environment because significant variance at the 1% level explained 69.25% of the total variation, while the genotype contributed 23.20% of the total variation of the experiment (Table 2). A large sum of squares for the environments indicated that the environments were diverse, with large differences among environmental means causing variation in the field emergence. The AMMI analysis revealed the complex nature of GEI and contributed 7.55% of the total sum of squares. The additional analysis of the GEI using the PCA analysis confirmed the statistical significance of the first two main component IPCA 1 and IPCA 2 (Table 1). Separately, IPCA1 and IPCA2 participated in the GE variation with 74.18% and 22.69% respectively, both with statistically significant effects on the GE interaction variation. These two main components jointly explained more than 96% of the variation of the genotype by environment interaction (Table 1).

**Figure 1:**
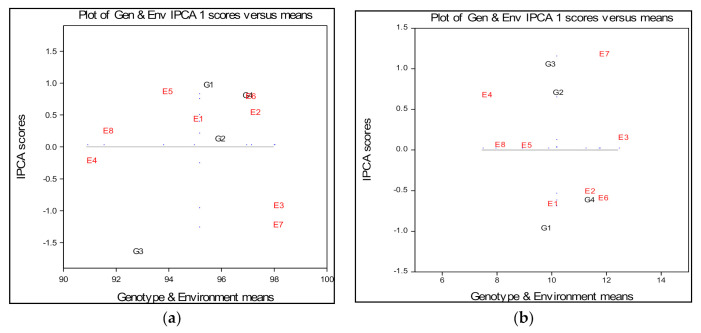
(**a**) Mean values of the field emergence of four wheat varieties grown in eight environments (2 years × 4 treatments), main and multivariate (genotype by environment interaction) effects; (**b**) Mean values of grain weight per plant (g) of four wheat varieties grown in eight environments (2 years × 4 treatments), main and multivariate (genotype by environment interaction) effects.

The combined analysis of variance showed that there are highly significant differences for the environment, the genotype and their interactions (Table 2). The AMMI1 biplot showed that genotype NK Ingenio (G3) had the largest distance from the average and at the same time this genotype had the highest distance from the IPCA1 (Figure 1a).

This can be explained by the fact that the emergence is very variable and this genotype can be considered to be very unstable in terms of seed priming. Small GEI values and a higher percentage of emergence than the average level, were achieved by genotype Pobeda (G1) and genotype Futura (G4) achieved the highest values of emergence. The greatest stability with IPCA scores close to zero and a higher yield than the average level was achieved by genotype NS40S (G2), as shown in Figure 1a. Observing the schedule of the genotypes compared to the different levels of seed priming, it can be concluded that across growing environments E2, E3, E6 and E7 the application of 10 mg L^−1^ and 100 mg L^−1^ ZnO NPs doses yielded the highest emergence for the most genotypes in both season (Figure 1a). These results clearly show that the percent of emergence differed between the cultivars, as well as by the priming concentration. However, despite the fact that certain ZnO NPs seed priming concentrations enhanced the plant emergence in the greenhouse, there is still no significant benefit for wheat emergence under field conditions. Hence, the seed priming of winter wheat still has limited value for promotion of seed emergence in field conditions, especially when wheat production has to meet with dry soil in the seed zone in conditions of limited precipitation, in wheat planting periods in which emergence is a major concern.

### 2.2. Grain Weight Per Plant

The greatest overall mean value for the grain weight per plant (13.40 g) was denoted for wheat variety Futura (G4) in the first year of study at 100 mg L^−1^ ZnO NPs applied. The same variety exhibited the highest mean value (13.15 cm) of grain weight per spike in the second year of trials at 10 mg L^−1^ ZnO NPs applied (Table 3). The same trend exposed the wheat genotypes Pobeda (G1), with maximum values (11.77 g) at 100 mg L^−1^ ZnO NPs applied in the first season, while in the second season the maximum values (11.79 g) were observed at 10 mg L^−1^ ZnO NPs. Genotypes NS40S (G2) and NK Ingenio (G3) showed the greatest values within the treatment at 100 mg L^−1^ ZnO NPs. The lowest values for grain weight per plant were found at the maximum concentration of ZnO NPs (Table 3).

Higher values of grain weight per plant were achieved under lower ZnO NPs concentration treatments (from 10 mg L^−1^ up to 100 mg L^−1^), while a reduced grain weight per plant was observed within the highest concentration of ZnO NPs applied. This variability was caused by the variability of the genetic materials and mostly the variability of the environmental conditions in which the wheat experiment was performed. The combined ANOVA showed that all three sources of variation (genotypes, treatments and environment) had significant influence on the phenotypic variation of trait grain yield per plant (Table 4). A large sum of squares for environments indicated that the environments were diverse, with large differences among the environmental means, causing the variation in the grain yields per plant. This result is in accordance with the results obtained by Rad et al. [28] and Mohammadi et al. [29]. The genotype by environment interaction expressed no significant mean square, leading to the conclusion that no cross interaction was expressed for the grain yield per plant. However, the additional analysis of the GEI using the PCA analysis showed thestatistical significance of the first source of variation, i.e. the first main component IPCA1, which participated in the GEI variation with 62.72% (Table 4).

According to the biplot (Figure 1b) and in terms of the average values (Table 3), it can be observed that agroecological environments E3, E5 and E8 are at the level of the experimental overall average. According to the arrangement of the E2, E3, E6 and E7 points, it can be concluded that the genotypes achieved higher average values of grain yield per plant in these environments compared to the E1, E5, E4 and E8 points. This result does not favor the E7 and E6 environments for obtaining higher values of grain weight per plant, given that the high values of interaction indicate the poor stability of this trait. Based on the graphic presentation, the interaction of the genotypes and environments, genotype Futura (G4), appeared to be better adapted on E2 and E6 environment, having mean value of grain yield per plant above the overall mean. Genotypes NS40S (G2) and NK Ingenio (G3) expressed a positive effect of interaction in the E3 and E7 environments, which correspond to environments within 100 mg L^−1^ ZnO NPs applied, keeping its average at the level of the overall mean. Genotype Pobeda (G1) appeared to be better adapted to the less favorable conditions of E1 environment keeping its average near to overall mean (Figure 1b).

### 2.3. Spike Length

Spike length is a genetically controlled trait, but it highly depends on environmental factors [28]. The results of this study showed that the spike length of wheat genotypes increased with the increasing ZnO NPs concentration applied until the maximum concentration of ZnO NPs has been applied. According to the results, wheat variety Futura (G4) has shown the highest average value for spike length (11.34 cm) in the first year of study at 10 mg L^−1^ ZnO NPs applied. The same variety exhibited the highest average value (11.38 cm) in a second year of study at 100 mg L^−1^ ZnO NPs applied at 10 mg L^−1^ ZnO NPs applied (10.83 cm). The lowest average value for the spike length (6.70 cm) was denoted for wheat variety Ingenio (G3) at 1000 mg L^−1^ ZnO NPs applied as in the second season within the same treatment (6.47 cm). The variation coefficient of spike length for all examined cultivars and years varied from 0.36% to 1.66% in the first year and from 0.34% to 1.42% in the second investigated year, with the maximum values at 1000 mg L^−1^ ZnO NPs applied (Table 5).

According to the analysis of variance of the AMMI model all of the sources of total variations (genotypes, treatments and environment) were statistically significant, with significant influence on the phenotypic variation. In the combined analysis of variance the main effects, the genotypes and environments were highly significant (36.56 + 87.54)/142.59 and explain 87.4% of the total variation. The participation of the genotype variation in the treatments sum of squares (SS) amounted to 25.6%, while 61.4% of the total sum of squares was attributable to environmental effects (Table 6).

A large sum of squares for the environments indicated differences between the growing seasons and diversity of treatments caused a considerable sum of squares for environmental factors in the total variation, indicating that these factors were the most responsible for the variation of the spike length. A higher influence of environmental factor on the spike length was also observed by Zečević et al. [30] and Mladenov et al. [31].

The genotype by environment interaction expressed a significant mean square, which suggests that the spike length of the genotypes varied across the environments. AMMI analyses revealed the complex nature of GEI and two statistically significant principal components were allocated. The first two main components jointly explained more than 98% of the variation of the genotype by environment interaction. The first source of variation, the quantified IPCA 1 axis, was also the largest and carried out about 77.9% of the total GE interaction sum of squares. The second principal component (IPCA2) contained about 21.6% of the sum of squares of the interaction (Table 6).

**Figure 2:**
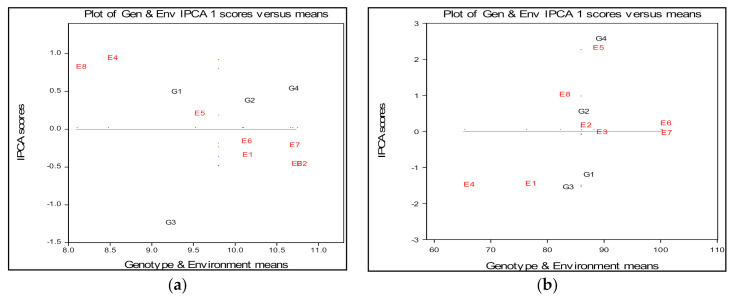
(**a**) Mean values of the spike length four wheat varieties grown in eight environments (2 years × 4 treatments), main and multivariate (genotype by environment interaction) effects (**b**) mean values of the plant height of four wheat varieties grown in eight environments (2 years × 4 treatments), main and multivariate (genotype by environment interaction) effects.

Significant interactions between environment and wheat cultivars in spike length, as high share of the first two main components IPCA1 and IPCA2 in the GE variation have been reported by Brbaklić et al. [32] and Mladenov et al. [31]. According to the biplot (Figure 2a) and in terms of the average values (Table 5), it can be noticed that, except for certain exceptions, all of the agroecological environments are quite near to the level of the experimental overall average. The small distance of the environmental points from the origin (zero point) indicates that the environments E5, E6, E7 and E1 were assessed as the most favorable to achieve a stable response of spike length of the wheat. According to the arrangement of the E5, E6, E7 and E1 points, it can be concluded that the cultivars achieved higher average values of spike length in these environments compared to the E4 and E8 points. Environments E4 and E8 had the highest interaction values, which makes them the least suitable for stable establishment spike of wheat. Genotypes NS40S (G2) and Futura (G4) were the most stable over all environments, indicating almost no cross interaction. Genotypes with above average means such as G2 (NS40S) and G4 (Futura) could be selected based on spike length, while genotype G3 (NK Ingenio) had a high distance from the average environment ordinate, exposed more variables and was less stable across the environments. The wheat variety Pobeda (G1) also showed stability for this trait with small GEI and responded well when grown in control variant environment. Varieties NS40S (G2) and G4 (Futura) responded favorable within treatments of 10 and 100 mg L^−1^ ZnO NPs (Figure 2a).

### 2.4. Plant Height

The results of this study showed that the plant height of wheat genotypes increased with the increasing ZnO NPs concentrations applied. The greatest overall mean value for plant height (91.16 cm) was denoted for wheat variety Futura (G4) in the first year of study at 100 mg L^−1^ ZnO NPs applied. The same variety exhibited the lowest mean value (75.05 cm) of plant height in the first year of trials at the variant of 1000 mg L^−1^ ZnO NPs applied (Table 7).

In the second vegetation season the greatest increases in plant height were found for the genotype Futura (103.01 cm) at 10 mg L^−1^ ZnO NPs and at the 100 mg L^−1^ ZnO NPs. Low values of plant height were observed at control plants, whereas the lowest values of plant height were found at the maximum concentration of ZnO NPs (Table 7). The presented results revealed that different treatments influenced the differences in plant height. Higher plant height was observed under lower ZnO NPs concentration treatments in the range between 10 and 100 mg L^−1^. On the other hand, reduced plant height was observed within the highest concentration of ZnO NPs applied. The plant height of wheat is a variable trait and its expression highly depends on the environmental factors. This was confirmed by high values of the coefficient of variation which ranged from 5.24% to 37.09% (Table 7). Some of the variability was caused by the variability of the genetic materials but mostly by the environmental conditions in which the wheat experiment was performed. The plant height of wheat is one of important yield components and is considered to be quantitative and variable trait the expression of which highly depends on the environmental factors [28].

The combined analyses of variance showed that all of the sources of total variations were statistically significant, having significant influence on the phenotypic variation of the plant height of the wheat. In the combined analysis of variance the main effects, genotypes and environments were highly significant ((420 + 11,403)/12,393) and explain 95.40% of the total variation. The participation of the genotype variation in the treatments sum of squares (SS) amounted to 3.34%, while 92.01% of the the total sum of squares was attributable to environmental effects. Differences of treatments caused a considerable sum of squares for environmental factors in the total variation which indicates that these factors were the most responsible for the variation of plant height. Four genotypes differed in their genotype by environment interaction, showing that genotype by environment interaction (GEI) was highly significant, as shown in Table 8.

The significant GEI indicated that the genotypes performance was inconsistent across testing environments. Additional analysis of the GEI interaction using the PCA (Interaction Principal Components) analysis revealed the statistical significance of the two main components, IPCA 1 and IPCA 2, which participated in the GEI variation with 60.11% and 28.47% respectively. These results indicate on genetic background of plant height, having major and minor genes in joint action, Table 8. This result is in accordance with the results obtained by Dimitrijević et al. [33].

In the AMMI biplot the genotype and environment main effects for plant height are presented on the *x*-axis, while the IPCA1 (Interaction Principal Component Axis 1) scores are on the *y*-axis (Figure 2b). The vertical line is the grand mean for the trait plant height and the horizontal line (y-ordinate) represents the IPCA1 value of zero. Small distance environmental points from the origin (zero point) indicate that E2, E3, E6 and E7 were assessed as the most favorable to achieve a stable reaction to plant height. Environments E4, E5, E1 and E8 had the highest interaction values, which determines them to be the least suitable for the stable establishment of the plant height of wheat.

The genotypes with above average means could be selected based on plant height, while a longer projection to the average environment ordinate demonstrates which genotypes are more variable and less stable across environments. Four wheat genotypes differed in genotype by environment interaction. Genotype NS40S (G2) was the most stable over all of the environments, indicating almost no cross interaction. On the contrary, the genotypes NK Ingenio (G3) and Futura (G4) showed the highest interaction at the level of the whole experiment. The wheat variety Pobeda (G1) has a smaller GEI than the previous genotypes and responded quite well when it was grown in a less favorable control variant environment. The variety NS40S (G2) responded favorably within treatments of 10 mg L^−1^ and 100 mg L^−1^ ZnO NPs, while the genotype Futura responded favorably within the control variant environment, giving the greatest value of the plant height (Figure 2b).

## 3. Materials and Methods

The present study was carried out at the experimental greenhouse facility at the University of Novi Sad, in Serbia, during two consecutive vegetation seasons of 2018/2019 and 2019/2020. Four varieties of hexaploid wheat (*Triticum aestivum* L.) namely, Pobeda (G1), NS40S (G2), NK Ingenio (G3) and Futura (G4) were selected and four different levels of zinc oxide nanoparticles were applied in the experiment in order to assess the variability in the yield related traits. Seeds of each wheat genotype were primed with different solutions containing appropriate concentrations of ZnO NPs (0 mg L^−1^, 10 mg L^−1^, 100 mg L^−1^ and 1000 mg L^−1^) for 48 h in a dark box by continuous aeration. The primed seeds were then sown in soil pots filled with 5.0 kg of soil, with 60–70% moisture contents during the whole experiment, untill the full maturity of the wheat. The trial was set up according to a completely randomized design, with three replications of each treatment on chernozem soil. The stability was followed within four levels of zinc oxide nanoparticles.

Each treatment in one growing season was considered to be a special environment. This produced eight different environment conditions of cultivation, which were in the same in agrotechnical terms, but different in their treatments of ZnO NPs seed priming (Table 9).

At the stage of full maturity, ten plants from each replication of each wheat genotypes were selected and their yield traits such as a field emergence (%), grain weight per plant (g), spike length (cm) and plant height (cm) were analyzed. At the beginning of the vegetation season, the field emergence (%) for each day was recorded, and the total and cumulative percent field emergence were determined relative to the number of seeds planted until a constant number of plants emerged. The genotype by environment interaction (GEI) was tested using the AMMI (Additive Main Effects and Multiplicative Interaction) analysis given by [32,33]. The AMMI model incorporates analysis of variance (ANOVA) and principal components analysis (PCA) into a single statistical model [15,34]. In the AMMI model, the ANOVA additive effects are separated from the interaction while, while additional GEI analysis can carried out by Principle Component Analysis (PCA) [31,35,36,37,38]. The biplot graphic presentation shows both the main and interaction effects for genotypes and environments simultaneously and provides a more in depth analysis of the G×E interaction [15,38,39]. The IPCA1 score of a genotype in the AMMI analysis was used as an indicator of the stability of a genotype over environments [29,39,40]. Zero IPCA value indicates highest stability, while an IPCA value long distance from zero indicates genotype instability.

The data processing was performed in GenStat 9th Edition (trial version) VSN International Ltd. (www.vsn-intl.com).

## 4. Conclusions

Based on the presented findings it can be concluded that seed priming with different concentrations of ZnO NPs possesses a great potential to improve all the examined traits of wheat in comparison with non-primed seed. The field emergence, grain weight per plant, spike length and plant height of wheat increased with the ZnO NPs concentration in the seed priming solution (up to 100 mg L^−1^) compared to the control. The maximum concentration of ZnO NPs in the priming solution decreased the mean values on an overall basis. The primed seeds of the wheat genotypes showed more uniform and better emergence, followed by higher crop growth during vegetation which provided better assimilation and distribution of dry matter and resulted in greater values of the observed traits. It was also evident that the wheat genotypes had various responses and they were greatly influenced by the combined effect of the varieties and ZnO NPs seed priming treatments. The overall results indicated that the examined yield traits of wheat were highly influenced by GEI effects and the magnitude of the environment effect showed a higher influence than the genotype effect for all of the observed traits. Regarding the field emergence, genotype NS40S was well adapted to the control conditions and treatment with a lower concentration of priming solution, yielding the smallest GEI. Genotypes Futura and Pobeda had the smaller interaction values, while the variety NK Ingenio responded well to the seed priming treatments. Regarding to the trait grain yield per plant, genotypes NK Ingenio and Pobeda appeared to be less stable, showing increased GEI, indicating a favorable response to the seed priming treatments. Genotypes Futura and NS40S reacted to different environments well, having the smallest GEI. Relating to the trait spike length, genotypes NS40S and Futura responded favorably within treatments of 10 mg L^−1^ and 100 mg L^−1^ ZnO NPs and could be selected based on spike length. The genotype NK Ingenio proved to be more variable and less stable across the environments, while stability for this trait also showed the variety Pobeda to have small GEI and it responded well in the control variants. With regard to the trait plant height, the genotype NS40S appeared to be more stable, with a small GEI, while genotypes Pobeda, NK Ingenio and Futura showed a more pronounced GEI and appeared to be less stable, indicating a favorable response to the seed priming treatments. In conclusion, this investigation indicates that the seed priming method might be an effective method for the improvement of important yield related traits of wheat and the estimation of the traits stability could provide valuable information to breeders and producers seeking to increase wheat productivity.

## Figures and Tables

**Table 1:** Mean values for field emergence (%) and interaction PCA1 values of the AMMI model of four wheat varieties grown in eight environments.

Field Emergence (%)							
Genotypes							
Environments	G1	G2	G3	G4	Em	IPCAe [1]	Variance
E1	95.44	95.57	91.79	96.86	94.92	0.37112	4.45
E2	97.71	97.74	93.79	99.11	97.09	0.47355	5.16
E3	97.27	98.54	97.16	98.92	97.97	−0.98531	1.28
E4	90.81	91.48	88.86	92.34	90.87	−0.28110	2.73
E5	94.67	94.42	89.90	96.01	93.75	0.80115	6.21
E6	97.75	97.57	93.18	99.11	96.9	0.72378	5.60
E7	96.98	98.50	97.66	98.68	97.95	−1.28690	0.60
E8	91.86	92.14	88.70	93.31	91.50	0.18372	4.82
IPCAg [1]	0.90567	0.05992	−1.70292	0.73733	95.12		10.68
Gm	95.31	95.75	92.63	96.79			

Gm: genotype mean; Em: environment mean; IPCAe [1]: Interaction principal component axes for environment; IPCAg [1]: Interaction principal component axes for genotype.

**Table 2:** AMMI analysis of variance for the field emergence of four winter wheat varieties examined across eight environments.

Source ^1^	df	SS	MS	F-Value	Share of Total Variation %
Total	95	1014.4	10.68	-	-
Treatments	31	974.9	31.45	65.77 **	96.11
Genotypes	3	226.2	75.42	157.73 **	23.20
Environments	7	675.1	96.45	93.32 **	69.25
Block	16	16.5	1.03	2.16 *	1.69
Interactions	21	73.6	3.5	7.33 **	7.55
IPCA [1]	9	54.6	6.07	12.7 **	74.18
IPCA [2]	7	16.7	2.39	4.99 **	22.69
Residuals	5	2.2	0.44	0.92 ^ns^	2.99
Error	48	23	0.48	-	-

^1^ All sources were tested in relation to the error; *: significant at the *p* < 0.05 probability level; **: highly significant at the *p* < 0.01 probability level; ns: not significant; df: degree of freedom; F: F value calculated; IPCA: interaction principal components axes.

**Table 3:** Mean values for grain weight per plant (g) and interaction PCA1 values of AMMI model of the four wheat varieties grown in eight environments.

Grain Weight Per Plant (g)							
Genotypes							
Environments	G1	G2	G3	G4	Em	PCAe [1]	Variance
E1	10.02	9.30	8.80	11.40	9.88	−0.71003	2.24
E2	11.22	10.74	10.25	12.62	11.21	−0.55240	2.10
E3	11.77	12.38	12.13	13.40	12.42	0.10500	1.45
E4	6.27	7.75	7.69	8.08	7.45	0.62931	1.04
E5	8.34	8.79	8.51	9.93	8.89	0.01144	1.08
E6	11.79	11.16	10.65	13.15	11.69	−0.63667	1.92
E7	10.04	12.37	12.48	12.02	11.72	1.13472	3.31
E8	7.36	7.83	7.55	8.96	7.92	0.01864	0.94
IPCAg [1]	−1.00917	0.65919	1.01033	−0.66035	10.14		4.792
Gm	9.60	10.04	9.75	11.19			

Gm: genotype mean; Em: environment mean; IPCAe [1]: Interaction principal component axes for environment; IPCAg [1]: Interaction principal component axes for genotype.

**Table 4:** AMMI analysis of variance for the grain weight per plant of four winter wheat varieties growing in eight environments.

Source ^1^	df	SS	MS	F-Value	Share of Total Variation %
Total	95	455.2	4.792	*	
Treatments	31	378.7	12.215	10.73 **	83.19
Genotypes	3	37.4	12.463	10.95 **	9.88
Environments	7	300.8	42.964	31.3 **	79.43
Block	16	22	1.373	1.21 ^ns^	5.81
Interactions	21	40.5	1.929	1.70 ^ns^	10.69
IPCA [1]	9	25.4	2.822	2.48 *	62.72
IPCA [2]	7	13.5	1.935	1.70 ^ns^	33.33
Residuals	5	1.6	0.313	0.28 ^ns^	0.42
Error	48	54.6	1.138	*	0.00

^1^ All sources were tested in relation to the error; *: significant at the *p* < 0.05 probability level; **: highly significant at the *p* < 0.01 probability level; ns: not significant; df: degree of freedom; F: F value calculated; IPCA [1]: the first interaction principal components axes; IPCA [2]: the second interaction principal components axes.

**Table 5:** Mean values for spike length (cm) and the interaction PCA1 values of the AMMI model of four wheat varieties grown in eight environments.

Spike Length (cm)							
Genotypes							
Environments	G1	G2	G3	G4	Em	IPCAe [1]	Variance
E1	9.36	10.28	9.95	10.75	10.083	−0.38314	0.36
E2	9.96	10.89	10.75	11.34	10.733	−0.50046	0.31
E3	9.90	10.83	10.69	11.28	10.675	−0.49779	0.38
E4	8.32	9.09	6.70	9.76	8.467	0.89740	1.66
E5	9.03	9.89	8.68	10.44	9.508	0.16308	0.63
E6	9.43	10.33	9.71	10.83	10.075	−0.20415	0.60
E7	9.98	10.89	10.35	11.38	10.65	−0.25478	0.34
E8	7.89	8.68	6.47	9.33	8.092	0.77983	1.42
IPCAg [1]	0.45054	0.33458	−1.27816	0.49304	9.785		1.58
Gm	9.233	10.108	9.162	10.637			

Gm: genotype mean; Em: environment mean; IPCAe [1]: Interaction principal component axes for environment; IPCAg [1]: Interaction principal component axes for genotype.

**Table 6:** AMMI analysis of variance for the spike length of four winter wheat varieties growing in eight environments.

Source ^1^	df	SS	MS	F-Value	Share of Total Variation %
Total	95	150.28	1.58	*	
Treatments	31	142.59	4.60	41.67 **	94.88
Genotypes	3	36.56	12.19	110.39 **	25.64
Environments	7	87.54	12.51	83.78 **	61.39
Block	16	2.39	0.15	1.35 ^ns^	1.68
Interactions	21	18.50	0.88	7.98 *	12.97
IPCA [1]	9	14.41	1.601	14.51 **	77.89
IPCA [2]	7	3.99	0.57	5.16 **	21.57
Residuals	5	0.1	0.02	0.18 ^ns^	0.07
Error	48	5.3	0.11	*	

^1^ All sources were tested in relation to the error; *: significant at the *p* < 0.05 probability level; **: highly significant at *p* < 0.01 probability level; ns: not significant; df: degree of freedom; F: F value calculated; IPCA [1]: the first interaction principal components; IPCA [2]: the second interaction principal components axes.

**Table 7:** Mean values for plant height (cm) and interaction PCA1 values of AMMI model of four wheat varieties grown in eight environments.

Plant Height (cm)							
	Genotypes						
Environments	G1	G2	G3	G4	Em	IPCAe [1]	Variance
E1	78.68	75.07	−−	75.05	76.08	−1.53868	7.17
E2	86.34	85.55	82.65	88.79	85.83	0.07406	9.61
E3	89.41	88.30	85.79	91.16	88.67	−0.11068	14.24
E4	67.80	64.14	64.68	64.06	65.17	−1.57010	5.24
E5	85.74	88.70	81.30	96.26	88.00	2.21890	37.09
E6	100.35	99.66	96.64	103.01	99.92	0.12898	20.99
E7	100.90	99.70	97.20	102.50	100.08	−0.13462	8.81
E8	81.48	82.20	77.49	87.16	82.08	0.93215	29.36
IPCAg [1]	−1.29245	0.45801	−1.63818	2.47262	85.73		135.38
Gm	86.33	85.42	82.67	88.5			

Gm: genotype mean; Em: environment mean; IPCAe [1]: Interaction principal component axes for environment; IPCAg [1]: Interaction principal component axes for genotype.

**Table 8:** AMMI analysis of variance for the plant height of four winter wheat varieties examined across eight environments.

Source ^1^	df	SS	MS	F-Value	Share of Total Variation %
Total	95	12,861	135.4	-	-
Treatments	31	12,393	399.8	52.17 **	96.36
Genotypes	3	420	140.2	18.29 **	3.39
Environments	7	11,403	1629	260.21 **	92.01
Block	16	100	6.3	0.82 ^ns^	0.81
Interactions	21	569	27.1	3.54 *	4.59
IPCA [1]	9	342	38	4.96 **	60.11
IPCA [2]	7	162	23.2	3.02 **	28.47
Residuals	5	65	13	1.7 ^ns^	11.42
Error	48	368	7.7	-	-

^1^ All sources were tested in relation to the error; *: significant at the *p* < 0.05 probability level; **: highly significant at the *p* < 0.01 probability level; ns: not significant; df: degree of freedom; F: F value calculated; IPCA [1]: first interaction principal components; IPCA [2]: second interaction principal components axes.

**Table 9:** Description of the eight different environments used to evaluate the four winter wheat genotypes.

Environments Code	Growing Season	Zno Nps Seed Priming Treatment
E1	2018/2019	0 mg L^−1^–control
E2	2018/2019	10 mg L^−1^
E3	2018/2019	100 mg L^−1^
E4	2018/2019	1000 mg L^−1^
E5	2019/2020	0 mg L^−1^–control
E6	2019/2020	10 mg L^−1^
E7	2019/2020	100 mg L^−1^
E8	2019/2020	L^−1^
